# Health related quality of life and its predictive factors on cervical cancer patients in two teaching hospitals, Addis Ababa, Ethiopia

**DOI:** 10.1186/s12905-024-03046-7

**Published:** 2024-04-02

**Authors:** Daniel Terefe Seyfu, Shiferaw Negash Abebe, Sofanit Haile, Birhanu Abera Ayana

**Affiliations:** 1 Department of Obstetrics and Gynecology, Yirgalem Hospital, Yirgalem, Ethiopia; 2 Department of Obstetrics and Gynecology, Addis Ababa University, Addis Ababa, Ethiopia; 3 University of Global Health Equity, Butaro, Rwanda

**Keywords:** Cervical cancer, Health-related quality-of-life, Predictive factors, Ethiopia

## Abstract

**Background:**

Cervical cancer is the second most prevalent and the leading cause of cancer related deaths among Ethiopian women; and about three fourth are diagnosed at advanced stages. Cervical cancer can affect the health-related quality of life (HRQOL) in multiple ways. The main aim of this study was to describe the HRQOL of cervical cancer patients and the predictive factors using validated tools.

**Methods:**

Institution based cross-sectional study was conducted among 264 cervical cancer patients using the validated Amharic version of European Organization for Research and Treatment of Cancer (EORTC) modules; QLQ-C30 and QLQ CX24. Descriptive statistics were used to summarize the raw data. One way ANOVA was used to determine the significance of mean differences between the dependent and independent variables. Binary and multivariable regression analysis were used to measure the association between Global Health Status and independent factors. The level of significance was set at *p*-value < 0.05.

**Results:**

On EORTC QLQ-C30 scales, the mean Global Health Status (GHS) was 42.57 ± 23.31. The least and highest affected functions were physical and social, mean (SD) = 76.39 ± 23.24 and 50.40 ± 32.19, respectively. The financial difficulty was the most affected among the symptom scales, 57.83 ± 35.34. Only physical function and financial difficulty have shown an independent association with GHS, (AOR = 0.21, 95% CI = 0.05–0.84), (AOR = 0.21 95% CI = 0.07–0.59), respectively. Illiterate, can read and write, were among the predictor factors that showed an independent association with the Global Health Status. Among the EORTC QLQ-CX24 symptom scales, the highest affected score was for sexual worry, mean (SD) = 51.81 + 32.197.

**Conclusions:**

In an effort to improve the Global Health Status of cervical cancer patients in Ethiopia; physical function and financial difficulty should be the priority areas. The Illiterate and those who lack formal education need due attention in order to improve the health-related quality-of-life.

**Supplementary Information:**

The online version contains supplementary material available at 10.1186/s12905-024-03046-7.

Aug 2022, Addis Ababa, Ethiopia.

## Introduction

Health-related quality-of-life (HRQOL) is a well-being that can be related to or affected by the presence of a disease or treatments. Quality-of-life (QOL) or Global quality-of-life of a person is an indication of a person’s well-being in ways of the ability to perform daily task, physical, emotional, cognitive, social, role and sexual functioning. It addresses the general perceptions of well-being as opposed to the specific functional scales. Quality-of-life (QOL) is a new dimension of care which has received greater attention in the last three decades [1]. Concern with HRQOL has grown as the life expectancy of cancer survivors has increased. Cervical cancer affects the HRQOL of women starting from diagnosis through treatment. It was noted that cervical cancer affects the body image and sexual activity of patients in addition to the social and physical functioning [2]. High levels of anxiety and depression are also observed in patients diagnosed with cervical cancer [3].

Cervical cancer was reported as one of the top three cancers affecting women of age younger than 45 years in 146 (79%) of the 185 countries assessed [4]. It is the commonest cancer in women in 28 countries and the commonest cause of cancer related deaths in 42 countries of the world; most being in Sub-Saharan Africa and South-East Asia [5]. It is the second most prevalent cancer next to breast cancer in Ethiopian women (TASH 2018, unpublished annual report of Clinical Oncology Department), and accounts for 9.3% and 10.2% of the cases and deaths in Ethiopia, respectively (WHO 2020). Many of these women with early-stage disease will be cured and have significant additional life expectancy following completion of treatment. Consequently, they will face years of potential treatment related side effects.

The high prevalence of the disease in Ethiopia calls for special attentions to patients, demanding action and public health priority. Determining HRQOL is associated with the survival benefit that a pharmacological treatment may provide. Despite the increasing prevalence and late stage at diagnosis of cervical cancer in Ethiopia [6], evidences are scarce with regards to the HRQOL of the cervical cancer patients. In addition, to the best of the authors’ knowledge, no study on the healthy-related quality-of-life of cervical cancer patients before the initiation of any treatment. The main aim of this study was to describe the HRQOL of cervical cancer patients and the predictive factors associated with it using the previously validated Amharic version of European Organization for Research and Treatment of Cancer module (EORTC QLQ C-30) and Cervical cancer module (EORTC QLQ-CX24) tools [7, 8] to provide a comprehensive understanding on the clinical status of cervical cancer patients attending the two referral hospitals, namely, Tikur Anbessa Specialized Hospital (TASH) and Saint Paul’s Hospital Millennium Medical College (SPHMMC). The availability of this data will assist health professionals involved in care provision to cervical cancer patients in Ethiopia to explore on the HRQOL domains of the disease and the treatment perspective; and impact the policy makers to design strategies to protect and improve the HRQOL of cervical cancer patients.

## Methods

### Study setting

The participants were recruited from the out-patient clinics and in-patient gynecology oncology services of TASH and SPHMMC, government owned, located in Addis Ababa, the capital city of Ethiopia, serving patients coming from all corners of the country. They are comprehensive and teaching hospitals with specialties and subspecialities in various fields. Each hospital has 6 gynecology oncologists, 4–6 gynecology oncology fellows, 80–120 residents in training in general gynecology and obstetrics, and about 30–40 registered clinical nurses in their respective Departments of Obstetrics and Gynecology. Each hospital has a capacity of 800 beds for in-patient services, of which one fourth is allocated for gynecology services, and around 35–40% of patients seen at gynecology referral clinics in these hospitals are cervical cancer cases. TASH also has Clinical Oncology Department providing service for over 60,000 patients annually. It is the core oncology referral and radiotherapy center for the entire country.

### Study design and period

A facility based cross-sectional study was conducted from Feb 1st to Aug 30, 2021. The data was collected from 264 eligible participants who visited the study sites during the study period. We used the combination of a generic quality-of-life questionnaire (EORTC QLQ-C30) and a specific questionnaire (EORTC QLQ CX24).

### Study participants

The study participants were all adult patients with histologically confirmed cervical cancer and ready to take the oncologic treatment (chemotherapy, radiotherapy and/or surgery) for the first time. The study variables were the sociodemographic and clinical characteristics of the participants, while the main outcome variables were different parameters of quality-of-life of cervical cancer patients. To avoid confounding; patients diagnosed with coexisting malignancies, HIV infections, psychiatric disorders and/ or those with serious communication problems; and those with severe medical conditions were excluded.

### Sample size and sampling procedure

The sample size (n) required for the study was calculated based on 5 to 10 patients per questionnaire item to generate stable reliability and validity estimates [9]. The tool has 24 items making the maximum sample size of 240, by considering the 10% non-response rate; the total sample size was 264 cervical cancer patients. Eligible participants were recruited and enrolled in the study as they appear for the clinical visit until the required sample size was achieved.

### Statistical analysis

Data was checked for completeness, cleaned, coded and then entered to SPSS version 25 for windows (SPSS, Inc. Chicago, USA). The scoring was based on the scoring manual provided by the quality-of-life coordinator at the EORTC Data Center [10]. The raw scores for both EORTC QLQ C-30 and EORTC QLQ-CX24 were computed, linear transformation of all scales and item scores ranging from 0 to 100 to obtain the standardized score was done [11]. Items representing one sub-scale were coded into their respective multi-item scales. The functional and GQOL scales were considered affected if the mean score was below 75, while for the affected symptom scales, the mean score was above 25. The higher scores indicate better functioning in the functioning scales and Global Health Status, but a higher level of difficulty for symptom scales and single symptom items.

The tool was easily understood with 100% compliance and with no missing responses. The internal consistency of the questionnaire was measured by the Cronbach’s α coefficient for each domain, the value calculated was 0.84 indicating good scale reliability [12].

Descriptive statistics were used to summarize the raw data. One way ANOVA was used to determine the mean differences between independent and the dependent variables. Bivariate and multivariable analysis were used to determine the association between the dependent variable (GQOL) and independent variables (functional and symptom scales, and predictor variables). The strength of association was measured by adjusted odds ratios and 95% confidence intervals with the level of significance set at *P*-value < 0.05.

## Results

### Socio-demographics and clinical characteristics of the study participants

Out of the 264 study participants, 154 (58.3%) were from TASH and the remaining, 110 (41.7%) were from SPHMMC. The age of the participants ranged from 25 to 81 years, with the mean (SD) of 51.8 ± 11.0 years. Majority, 201 (76.1%) of the participants were in the age group of 40–69 years and 143 (54.2%) of the participant were married and 59.9% of the participants came from the two largest regions of the country, Oromia and Amhara, and only 14.8% of the participants were from the capital city, Addis Ababa. One hundred twenty (44.3%) of the participants had no formal education. The average monthly household income for the majority, 157 (59.5%) of the participants was above poverty line for the country, ≥ 6000 birr/month (1USD = 46.32 birr for Sep 2021) (Table 1).

**Table 1:** Socio-demographic characteristics of the study participants, Aug 2021

Variables	Category	Frequency	Percent
Age in years	< 40	44	16.7
	40–49	57	21.6
	50–59	89	33.7
	60–69	55	20.8
	> 70	19	7.2
Region	Tigray	11	4.2
	Afar	8	3.0
	Amhara	67	25.4
	Oromia	91	34.5
	Somalia	6	2.3
	Benishangul	2	0.8
	SNNPR*	34	12.9
	Harari	1	0.4
	Addis Ababa	39	14.8
	Dire Dawa	5	1.9
Religion	Orthodox	132	50.0
	Muslim	79	29.9
	Protestant	53	20.1
Educational status	Illiterate	117	44.3
	Can read and write	48	18.2
	Informal education	33	12.5
	Primary	21	8.0
	Secondary	6	2.3
	College and above	39	14.8
Marital Status	Single	14	5.3
	Married	143	54.2
	Divorced	40	15.2
	Widowed	67	25.4
Occupational Status	Government employee	34	12.9
	Private employee	21	8.0
	Merchant	26	9.8
	Retired	5	1.9
	Farmer	47	17.8
	Housewife	129	48.9
	Unemployed	2	0.8
Monthly Income	< 6000 ETB	107	40.5
	≥ 6000ETB	157	59.5

*SNNPR- Southern Nations Nationalities Peoples Regional State

Most (73.8%) of the cases were at FIGO (The International Federation of Gynecology and Obstetrics) stage II and III at the time of enrollment. The majority (90.5%) of the participants were diagnosed within one year of the data collection period. Chemoradiation (51.5%) followed by surgery (34.8%) were the two most planned treatments, respectively. Most (84.5%) of the participants did not have comorbid conditions (Table 2).

**Table 2:** Clinical characteristics of the study participants, Aug 2021

Variables	Category	Frequency	Percent
Patient status	New patient	208	78.8
	On follow up	56	21.2
Stage of cancer	stage I	41	15.5
	stage II	92	34.8
	stage III	103	39.0
	stage IV	28	10.6
Time since diagnosis	< 1yr	239	90.5
	1-5yrs	24	9.1
	> 5yrs	1	0.4
Current treatment plan	Surgery	92	34.8
	Chemotherapy	27	10.2
	Radiation therapy	2	0.8
	Chemo-radiation	136	51.5
	Chemotherapy followed by surgery	7	2.7
Comorbidity condition	None	223	84.5
	HTN	21	8.0
	DM	11	4.2
	Cardiac	2	0.8
	Renal	1	0.4
	Others	6	2.3

### EORTC QLQ-30 and EORTC QLQ-CX24

Cervical cancer has a major effect on a patient’s health-related quality-of-life which includes physiological and psychological impact. It was noted that cervical cancer affects the body image and sexual activity of patients in addition to the social and physical functioning. High levels of anxiety and depression are also observed in patients with cervical cancer. As shown in Table 3, the Global Health Status/Global quality-of-life (GQOL) mean (SD) score was 42.57 ± 23.31. The EORTC QLQ-C30 functional scales mean (SD) ranged from 50.4 ± 32.2 to 76.4 ± 23.2 with the least affected being physical function and highest affected being social function. The financial difficulty had the highest mean (SD) score of 57.8 ± 35.3; and with the exception of diarrhea 20.08 ± 29.87 and dyspnea 22.89 ± 29.87, all the other items indicated moderate to high symptoms. The EORTC QLQ-CX24 items exhibited a range of mean (SD) scores from 24.5 ± 28.1 for the sexual enjoyment to 49.4 ± 24.1 for the sexual activity. The least affected symptom was Lymphedema, mean (SD) 27.7 ± 37.1 and highest affected was for sexual worry 51.8 + 32.2.

**Table 3:** Description of the Global Health Status, functional, and symptom scales among cervical cancer patients, Aug 2021

Variables	Item numbers	Mean	standard deviation (SD)
**Global QOL (Global health status)**	29,30	42.6	23.3
**Functional scales**			
Physical function	1–5	76.4	23.2
Role function	6,7	72.5	27.6
Emotional function	21–24	63.9	23.4
Cognitive function	20,25	72.5	25.0
Social function	26,27	50.4	32.2
**Symptom scales**			
Fatigue	10,12,18	40.7	23.8
Nausea and vomiting	14,15	30.5	31.3
Pain	9,19	36.8	23.7
Dyspnea	8	22.9	29.9
Insomnia	11	42.2	28.1
Loss of appetite	13	40.2	34.4
Constipation	16	32.1	37.3
Diarrhea	17	20.1	29.9
Financial difficulty	28	57.8	35.3
Variables (EORTC QOL-24)	Item numbers	Mean	SD
**Functional scales**			
Body image	45–47	45.7	28.9
Sexual and vaginal	50–53	38.9	20.8
Sexual activity	49	49.4	24.1
Sexual enjoyment	54	24.5	28.1
**Symptom scales**			
Symptom experience	31–34,39,41–43	35.0	22.3
Lymphedema	38	27.7	37.1
Peripheral neuropathy	40	32.5	37.5
Menopausal symptoms	44	30.5	36.9
Sexual worry	48	51.8	32.2

The association of the sociodemographic and clinical characteristics of cervical cancer patients with EORTCQLQ-C30 and EORTCQLQ-CX24; Global Health Status, specific functional, and symptom scales have been presented and described in the succeeding tables and paragraphs [S1-8 Tables] using one way ANOVA (See supplement). In our study, marital status showed significant association with social function, *p*-value 0.014, while occupation showed significant association with Global Health Status and social function, *p*-value 0.000 and 0.005, respectively. Those participants who learnt up to college and above had more affected GQOL and role functions [S1 Table].

There was no significant mean difference across the functional scales and comorbid conditions, but the time of encounter (patient being new or on follow-up awaiting treatment) showed significant association with most functional scales except the GQOL and social function. However, participants with FIGO stage I cervical cancer scored a significantly higher mean in Global Health Status and in all functional scales. All functional scales of patients with FIGO stage IV cervical cancer were found to have the lowest means. The stage of cervical cancer and current planned type of treatment had significant mean difference in all functional scales. The more advanced the stage of cervical cancer, the more the Global Health Status affected. Patients whose current plan of treatment is radiotherapy showed the least mean for GQOL mean (SD) = 16.67 ± 0.00, and role functioning mean (SD) = 16.67 ± 23.57, while patients who were planned to have surgery as current planned treatment showed the highest mean for all functional scales except GQOL [S2 Table].

The mean difference of all symptom scales was significant across the age groups except financial difficulty. Marital status showed significant mean difference with constipation and financial difficulty in the EORTC QLQ-C30 module. Similarly, the widowed groups showed the highest mean difference with fatigue, constipation, and financial difficulty. The educational status exhibited a significant mean difference with constipation scale. Participants who are illiterate had higher mean scores with fatigue and financial difficulty as compared to other educational status categories. Those who are unemployed have the lowest mean difference scores across all symptom scales except financial difficulty [S3 Table].

FIGO disease stages, time since diagnosis, and the current planned type of treatment options indicated significant mean differences across all or most EORTC QLQ C30 symptom scales. However, no significant difference was observed for comorbid conditions [S4 Table].

Age has shown a significant mean difference in the body image and sexual activity. Participants in the age category of 60–69 years have the highest mean scores in all functional scales. Marital status had significant mean difference score with the sexual activity. Education and occupation have revealed significant mean differences with body image. The highest mean score was reported in those learnt up to college and above mean (SD) = 52.99 ± 25.29. A monthly household income did not exhibit a significant difference in the scales except in body image [S5 Table].

As depicted in S6 Table, time since diagnosis and the stage of disease have shown significant association with body image, *p* < 0.000 and *p* < 0.023, respectively. Stage IV disease has the highest mean score in sexual and vaginal function, mean (SD) = 60.42 ± 22.160. Participants whose plan of treatment was radiation therapy had the highest mean score for the body image, mean (SD) = 61.11 ± 39.284.

On the analysis of the EORTC QLQ-CX24 symptom scales; age was shown to have significant association with symptom experience, lymphedema, and sexual worry while marital status showed a significant association with symptom experience only. Participants falling in the age group of 70 years and above showed the highest mean difference in all symptom scales. Those who are widowed scored highest mean difference across symptom experience and menopausal symptoms mean (SD) = 40.03 ± 16.76 and 43.28 ± 41.04, respectively. Educational status showed significant mean difference with symptom experience, menopausal symptoms, and sexual worry. Those who learnt up to college and above had the highest mean score in the menopausal symptom scales, mean (SD) = 58.12 ± 41.68. Occupational status had significant mean difference with symptom experience and lymphedema. Those who were retired from job had the highest mean difference across lymphedema scales, mean (SD) = 86.67 ± 29.814. Monthly income of participants indicated a significant mean difference among lymphedema and menopausal symptom groups [S7 Table].

The patients’ status exhibited a significant mean difference with the symptom experience, lymphedema and menopausal symptoms. Time since diagnosis, stages of the disease, and planned types of treatments showed significant mean difference across all symptom scale except sexual worry. Those diagnosed to have stage IV cervical cancer scored the highest mean score in all symptom scales. Those whose planned treatment type was radiation have the highest mean scores in all symptom scales except sexual worry. Comorbid conditions revealed a significant mean difference with lymphedema and peripheral neuropathy. Participants who had cardiac disease concomitant with cervical cancer exhibited the highest mean difference in the peripheral neuropathy, mean (SD) = 83.33 ± 23.570 [S8 Table].

### Predictive factors of global quality-of-life

Tables 4 and 5 detail the association of the predictive factors, the specific functional, and symptom scales of EORTC QLQ C30 with Global Health Status. During the bivariate analysis, all variables with *p*-value < 0.25 were included for the multivariable logistic regression. Among the sociodemographic variables; illiterate (COR = 0.12 95% CI = 0.03–0.41, AOR = 0.79 95% CI = 0.01–0.65)), can read and write (COR = 0.15 95% CI = 0.03–0.72, AOR = 0.85 95% CI = 0.01–0.91), respectively have shown an independent association with GQOL. Whereas, being a house wife was significant with Global Health Status only on bivariate model (Table 4).

**Table 4:** Binary and multivariable logistic regression analysis of socio-demographic variables with GQOL of patients with cervical cancer, Aug 2021

Variables	Category	GQOL		Odds Ratio (95% CI)	
		Affected	Not affected	COR	AOR
Age	< 40	42 (17.3%)	2 (9.5%)	3.12(0.44–27.9)	
	40–49	52(21.4)	5(23.8%)	4.39(0.53–36.4)	
	50–59	80(32.9%)	9(42.9%)	1	
	60–69	50(20.6%)	5(23.8%)	1	
	> 70	19(7.8%)	0	1	
Education status	Illiterate	113(46.5%	4(4.8%)	0.12(0.034–0.41)	0.08(0.01–0.72)
	Can read and write	46(18.9%)	2(9.5%)	0.15(0.03–0.71)	0.80(0.01–0.83)
	Informal education	33(13.6%)	0	0	0
	Primary	18(7.4%)	3(14.3%)	0.56(0.13–2.32)	
	Secondary	3(1.2%)	3(14.3%)	3.33(0.57–19.47)	
	College and above	30(12.3%)	9(42.9%)	1	
Occupational Status	Government employee	27(11.1%)	7(33.3%)	1	
	Private employee	19(7.8%)	2(9.5%)	0.41 (0.08–2.17)	
	Merchant	22(9.1%)	4(19.0%)	0.70(0.18–2.71)	
	Retired	5(2.1%)	0	0.00	
	Farmer	47(19.3%)	0	0.00	
	Housewife	121(49.8%	8(38.1%)	0.26(0.09–0.76)	
	Unemployed	2(0.8%)	0	0.000	
Monthly income	< 6000ETB	101(41.6%)	6(28.6%)	0.56(0.21–1.50)	
	≥ 6000ETB	142(58.4%)	15(71.4%)	1	

On bivariate model, no statistically significant association was observed between the clinical characteristics (time since diagnosis, stage of cancer, and treatment plan) and the Global Health Status. However, participants with time since diagnosis ≥ 1 year were 3 times likely to have an affected GQOL as compared to those < 1 year (COR = 1.05(0.23–4.82), AOR = 3.01(0.53–16.91).

On the analysis of EORTC QLQ-C30 functional scales of patients with cervical cancer, only physical function has shown an independent association with the Global Health Status, (COR = 0.33, 95% CI = 0.12–0.64, AOR = 0.21, 95% CI = 0.05–0.84).

Pain and appetite have shown significant association with Global Health Status on the bivariate model, pain (COR = 0.34 95% CI = 0.14–0.83), appetite (0.34 95% CI = 0.14–0.83), respectively. However, only financial difficulty exhibited an independent association with GQOL, (COR = 0.15 95% CI = 0.05–0.39, AOR = 0.21 95% CI = 0.07–0.59), respectively (Table 5).

**Table 5:** Binary and multivariable logistic regression analysis of the EORTC QLQ-C30 scales with GQOL of patients with cervical cancer, Aug 2021

Variables	Category	Outcome	GQOL		Odds Ratio(95%CI)	
			Affected	Not affected	COR (95%CI)	AOR (95%CI)
Functional scales	Physical	Affected	117(48.1%)	5(23.8%)	0.33(0.12–0.64)	0.21(0.05–0.84)
		Not affected	126(51.9%)	16(76.2%)	1	
	Role	Affected	126(51.9%)	10(47.6%)	0.84(0.34–2.06)	
		Not affected	117(48.1%)	11(52.4%)	1	
	Cognitive	Affected	114(46.9%)	7(33.3%)	0.56(0.22–1.45)	
		Not affected	129(53.1%)	14(66.7%)	1	
Symptom scales	Fatigue	Affected	180(74.1%)	13(61.9%)	0.56(0.22–1.43)	
		Not affected	63(25.9%)	8(38.1%)	1	
	Nausea &Vomiting	Affected	114(46.9%)	6(28.6%)	0.45(0.17–1.20)	
		Not affected	129(53.1%)	15(71.4%)	1	
	Pain	Affected	177(72.8%)	10(70.8%)	0.34(0.14–0.83)	0.60(0.16–2.27)
		Not affected	66(27.2%)	11(52.4%)	1	
	Dyspnea	Affected	99(40.7%)	7(33.3%)	0.72(0.28–1.86)	
		Not affected	144(59.3%)	14(66.7%)	1	
	Appetite	Affected	195(80.2%)	11(52.4%)	0.27(0.10–0.67)	0.52(0.16–1.64)
		Not affected	48(19.8%)	10(47.6%)	1	
	Constipation	Affected	162(66.7%)	11(52.4%)	0.55(0.22–1.34)	
		Not affected	81(33.3%)	10(47.6%)	1	
	Diarrhea	Affected	66(27.2%)	4(19%)	0.63(0.20–1.94)	
		Not affected	177(72.8%)	17(81%)	1	
	Financial	Affected	218(89.7%)	12(57.1%)	0.15(0.05–0.39)	0.21(0.07–0.59)
		Not affected	25(10.3%)	9(42.9%)	1	1

One of the interesting findings of this study was that none of the functional and the symptom scales of EORTC QLQ CX 24 have shown a statistically significant association with GQOL when assessed on binary and multivariable logistic regression analysis.

## Discussion

The mean score for GQOL of cervical cancer patients in our study was low indicating poor QOL. The functioning domain of EORTCQLQ 30 and EORTCQLQ CX 24 resulted in a score lower than the reference values except in role function. All the symptom scales were higher than the reference values placed by the EORTC group [13]. Illiterate and those who can read and write portrayed among the predictor factors an independent association with Global Health Status.

The low mean score (42.6 ± 23.3) for GQOL of cervical cancer patients in our study is far lower than the EORTC QLQ reference value manual for cervical cancer patients (60.0 ± 25.2) indicating poor QOL [14]. These differences could be partly explained by the late stage at diagnosis and the patients’ assumption of an exaggerated symptom report will entail more attention from the health care professional. Similar to the studies from Tanzania and Indonesia, financial difficulty was the worst affected QOL dimension among the symptom scales in our patients [15, 16]. Contrary to the Turkish and Malaysian studies, where either pain or fatigue were affected the most, our study showed both symptom domains were similarly affected [17, 18]. This could be due to the inaccessibility of the oncology services in Ethiopia whereby patients need to travel long distances, which make both fatigue and pain to have been affected at the same time.

Similar to the result from Turkey, educational status has shown a significant association with HRQOL components [17]. The findings of the lower score in social function among the house wives in our study is supported by the study from the Sudan, where higher GQOL scores were reported for patients who are employed in medium and high skill occupation [19].

Social and sexual functioning were found to have a minimum score among the functional scales, which mirrors previous findings, where patients with cervical cancer found it difficult to interact with their community and engage in sexual activity due to the illness and treatment [7]. Sexual activity is among the least reported functioning item in most groups of patients with cervical cancer. The slightly higher sexual activity proportion reported in our participants, 31.4%, could possibly be explained by the majority of participants being young (< 40 years) and most being married as these situations may increase the opportunity for sexual exposure.

Similar to the finding from India, diarrhea was among the least reported symptoms in our participants whereas diarrhea in cervical cancer patients was commonly described as a side effect to medical treatments [20]. Comparable to the study in Brazil, having a current occupation was associated with better HRQOL in our participants [21].

A significantly high mean score in functional domains and low symptom scales in our FIGO stage I cancer patients conform to the results from other studies where higher scores for physical and role functioning with patients at earlier stages of cervical cancer [8, 22]. In patients with FIGO stage IV, the ability to interact with their community and participate in household tasks demonstrated to have declined due to the prevalence of symptoms and lower functionality scores; which can be reflected directly on the role and social functioning.

Physical function is an often neglected but integral part of the HRQOL of patients [23]. In Ethiopia, where women play an immense role in the household, the physical function ability might have a great impact on the self-satisfaction and HRQOL of patients [24].

Fatigue and dyspnea in the EORTC QLQ-C30 scale and lymphedema from the EORTCQLQ-CX24 in our study were reported to have higher mean scores. Reports from other studies underlined the degree of pain and symptom experience directly affected the GQOL of patients with cervical cancer [25, 26].

Emotional distress is a strong factor behind the battle with cervical cancer and patients with cervical cancer reported to have a higher amount of anxiety and depression [3, 27]. The conflicting finding in our study that emotional functioning has no significant association with GQOL could possibly be explained by the cultural inhibition on the image of an illness that could impact on the HRQOL of our subjects [22, 28].

Social support and sexual function were reported as predictive of the GQOL of patients [29–31]; our study did not demonstrate such association. More attention on the impact of the illness on the general health than the emphasis given to sexual activity in our patients might explain such disparity.

## Limitations

The relatively large sample size, the use of validated measurement tools, and the cultural, ethnic, and religious diversity of the study participants are some of the strengths of this study. To the best our knowledge, the health-related quality-of-life of cervical cancer patients before initiation of any treatment is the first of its kind in our country. Since all of our study participants were within 5 years since diagnosis, we can’t generalize to the entire patients who survived more than 5 years.

## Conclusions

The disease has significantly affected the HRQOL of our participants as demonstrated by far the very low mean score for GQOL and higher mean scores for most symptom scales in both modules. An approach with emphasis on the physical function, financial difficulty, and education will improve the Global Health Status and the HRQOL of cervical cancer patients in Ethiopia.

The incorporation of the qualitative study to better understand the patients spiritual and emotional connection is recommended.

### Electronic supplementary material

Below is the link to the electronic supplementary material.

Supplementary Material 1

## Data Availability

All data generated or analyzed during this study are included in this published article [and its supplementary information files].

## Abbreviations

EORTC QLQ-C30: European Organization for Research and Treatment of Cancer Quality-of-life Questionnaire Core-30
EORTC QLQ-CX24: European Organization for Research and Treatment of Cancer Quality-of-life Questionnaire- cervical cancer module
GHS: Global Health Status
QOL: quality-of-life
HRQOL: Health-related quality-of-life
TASH: Tikur Anbassa Specialized Hospital
SPHMMC: Saint Paul’s Hospital Millennium Medical College
