# Supplementary material for: Health related quality of life and its predictive factors on cervical cancer patients in two teaching hospitals, Addis Ababa, Ethiopia
Source: BMC Womens Health. 2024 Apr 2;24:209. doi: 10.1186/s12905-024-03046-7 (PMC10986003; doi:10.1186/s12905-024-03046-7)
Supplement: Supplementary file 1 — Supplementary Material 1 [file 12905_2024_3046_MOESM1_ESM.docx]

**Supporting/supplementary file**

**This is S1 Table: Analysis of the mean differences of** **EORTCQLQ-C30 functional scales with socio-demographic characteristics using one way ANOVA, Aug 2021.**

| Variables | Category | GQOL | PF | RF | EF | CF | SF |
| --- | --- | --- | --- | --- | --- | --- | --- |
| Age | <40 | 40.34±23.08 | 82.88±19.41 | 79.92±27.74 | 66.29±22.51 | 79.17±24.14 | 50.76±29.84 |
|  | 40-49 | 44.30±24.45 | 74.62±22.65 | 66.37±30.45 | 65.50±23.7 | 73.68±24.38 | 48.25±31.76 |
|  | 50-59 | 40.82±23.70 | 77.00±21.24 | 69.29±33.13 | 62.83±20.5 | 74.91±26.84 | 43.26±29.70 |
|  | 60-69 | 41.36±23.56 | 64.61±25 | 63.03±34.05 | 59.24±23.0 | 66.67±29.04 | 36.67±30.66 |
|  | ≥70 | 25.88±17.76 | 55.09±22.53 | 57.02±31.58 | 54.39±19.3 | 61.40±29.42 | 36.84±31.22 |
|  | p-value | 0.221 | 0.000* | 0.000* | 0.193 | 0.051* | 0.114 |
| Marital status | Single | 50.60±27.04 | 82.38±15.15 | 84.52±23.98 | 72.02±20.8 | 80.95±17.11 | 64.29±32.59 |
|  | Married | 39.98±22.80 | 73.15±24.43 | 70.28±32.14 | 62.82±22.2 | 73.43±26.84 | 44.06±29.89 |
|  | Divorced | 41.88±25.70 | 75.50±23.06 | 64.58±32.28 | 62.37±22.2 | 69.58±27.70 | 47.50±31.24 |
|  | Widowed | 38.81±23.23 | 70.45±22.36 | 62.69±32.70 | 59.2±21.81 | 71.14±28.06 | 36.57±29.90 |
|  | p-value | 0.376 | 0.325 | 0.82 | 0.223 | 0.536 | 0.014 |
| Educational status | Illiterate | 33.26±20.89 | 72.31±23.53 | 68.66±32.02 | 61.68±22.77 | 74.50±27.38 | 38.75±29.83 |
|  | Can read and write | 35.76±21.87 | 73.47±22.66 | 72.9 2±27.42 | 61.81±20.4 | 74.65±24.30 | 46.87±29.90 |
|  | Informal education | 41.16±21.74 | 82.63±17.71 | 79.29±23.21 | 58.33±22.14 | 75.25±24.33 | 45.96±36.33 |
|  | Primary | 52.38±16.69 | 73.97±26.06 | 72.22±30.88 | 69.84±20.15 | 75.40±23.34 | 47.62±28.03 |
|  | Secondary | 70.83±29.69 | 74.44±25.79 | 58.33±41.83 | 70.83±22.82 | 77.78±27.21 | 66.67±40.82 |
|  | Collage and above | 56.62±24.27 | 67.69±25.06 | 51.28±38.30 | 64.96±23.11 | 60.26±30.01 | 47.44±27.45 |
|  | p-value | 0.000* | 0.169 | 0.005* | 0.417 | 0.077 | 0.154 |
| Occupational status | Government | 53.68±25.72 | 70.98±25.84 | 59.31±39.60 | 61.76±22.01 | 63.73±27.63 | 47.06±31.64 |
|  | Private | 44.05±24.74 | 78.41±16.85 | 74.60±28.68 | 71.43±19.28 | 73.81±27.67 | 65.08±25.22 |
|  | Merchant | 53.85±22.14 | 72.82±26.79 | 63.46±35.90 | 63.14±22.75 | 75.00±21.21 | 46.15±30.29 |
|  | Retired | 45.00±21.73 | 66.67±21.08 | 46.67±38.00 | 53.33±27.38 | 46.67±36.13 | 40.00±30.27 |
|  | Farmer | 31.91±21.01 | 74.61±23.20 | 71.99±32.98 | 63.12±23.87 | 74.11±28.40 | 44.33±27.20 |
|  | Housewife | 36.82±21.91 | 72.56±23.16 | 69.64±28.97 | 61.18±21.66 | 74.81±26.19 | 39.02±31.62 |
|  | Unemployed | 37.50±17.67 | 100±000 | 91.67±11.78 | 83.33±000 | 75.00±35.35 | 33.33±000 |
|  | p-value | 0.000* | 0.594 | 0.224 | 0.362 | 0.138 | 0.027 |
| Monthly income | <6000ETB | 33.02±21.565 | 75.20±21.55 | 73.83±28.96 | 66.2±20.63 | 80.84±22.173 | 40.34±31.89 |
|  | ≥6000ETB | 45.65±23.619 | 72.02±24.488 | 64.44±33.76 | 60.19±22.85 | 67.09±23.367 | 46.34±29.72 |
|  | p-value | 0.000* | 0.277 | 0.020 | 0.030 | 0.000* | 0.137 |

PF: Physical functioning, EF: Emotional functioning, RF: Role functioning, CF: Cognitive functioning, SF: Social functioning, *p<0.05

**This is S2 Table: Analysis of the mean differences of EORTC QLQ-C30 functional scale with clinical characteristics using one way ANOVA, Aug 2021**.

| Variables | Category | GQOL | PF | RF | EF | CF | SF |
| --- | --- | --- | --- | --- | --- | --- | --- |
| Patient status | New | 40.02±23 | 75.48±23.1 | 73.00±28.91 | 64.02±21.3 | 75.24±25.21 | 45.03±31.28 |
|  | Follow up | 42.41±25.53 | 65.24±22.3 | 50.60±37.4 | 57.44±24.3 | 63.10±30.6 | 38.99±28.11 |
|  | p-value | 0.503 | 0.003* | 0.000* | 0.048 | 0.003* | 0.191 |
| Time since Dx | <1yr | 40.55±23.13 | 74.76±22.94 | 71.27±30.74 | 63.74±21.87 | 75.03±26.05 | 44.70±30.63 |
|  | 1-5yrs | 41.32±28. | 60.56±23.0 | 40.97±31.46 | 53.13±21.9 | 51.39±23.52 | 34.72±31.05 |
|  | p-value | 0.593 | 0.004* | 0.000* | 0.019 | 0.000* | 0.299 |
| Stage of cancer | Stage I | 53.66±25.21 | 85.53±21.12 | 85.37±22.42 | 70.33±20.58 | 82.52±20.05 | 62.20±28.63 |
|  | Stage II | 43.03±20.72 | 81.23±19.66 | 78.44±25.62 | 68.30±21.83 | 81.34±24.06 | 51.45±29.61 |
|  | Stage III | 37.22±23.73 | 68.09±20.44 | 64.08±30.13 | 58.74±20.69 | 67.96±25.74 | 33.50±27.95 |
|  | Stage IV | 25.30±18.19 | 48.57±24.26 | 25.00±29.91 | 47.02±20.31 | 47.02±28.34 | 29.17±26.69 |
|  | P-value | 0.000* | 0.000* | 0.000* | 0.000* | 0.000* | 0.000* |
| Treatment plan | Surgery | 49.46±23.59 | 84.20±19.20 | 83.33±21.54 | 69.20±20.36 | 82.97±20.52 | 55.98±30.32 |
|  | Chemo | 41.05±20.27 | 67.65±21.69 | 65.43±30.28 | 62.65±27.46 | 67.90±29.20 | 42.59±31.80 |
|  | Rad | 16.67±.00 | 60.00±9.42 | 16.67±23.57 | 66.67±35.35 | 66.67±.00 | 33.33±.00 |
|  | Chemoradiation | 34.25±22.58 | 66.57±23.92 | 58.70±34.89 | 57.60±21.06 | 65.81±28.49 | 36.03±28.41 |
|  | NAC with surgery | 50.00±17.34 | 86.67±10.88 | 80.95±14.99 | 72.62±19.07 | 90.48±8.90 | 40.48±33.13 |
|  | p-value | 0.000* | 0.000* | 0.000* | 0.002* | 0.000* | 0.000* |
| Comorbid conditions | None | 41.63±24.15 | 73.99±23.77 | 68.61±32.63 | 63.12±21.73 | 73.02±26.90 | 45.37±30.60 |
|  | HTN | 38.49±23.78 | 69.84±20.19 | 67.46±29.09 | 69.44±23.61 | 73.02±25.53 | 42.06±33.17 |
|  | DM | 32.58±6.92 | 64.85±24.05 | 66.67±28.86 | 55.30±19.46 | 69.70±27.70 | 33.33±29.81 |
|  | cardiac | 41.67±11.78 | 63.33±32.99 | 66.67±47.14 | 62.50±29.46 | 66.67±23.57 | 25.00±11.78 |
|  | Renal | 16.67 | 66.67 | .00 | 41.67 | 66.67 | 33.33 |
|  | *Others | 25.00±19.72 | 80.00±15.77 | 72.22±27.21 | 37.50±21.57 | 66.67±38.00 | 16.67±18.25 |
|  | p-value | 0.358 | 0.698 | 0.461 | 0.034 | 0.987 | 0.187 |

*Others: anemia, epilepsy, peptic ulcer disease, p-value<0.005. NAC-neoadjuvant chemotherapy

**This is S3 Table: Analysis of the mean differences in EORTCQLQ-C30 symptom scale with socio-demographic characteristics using one way ANOVA, Aug 2021**.

| Variables | Category | FA | NV | PA | DY | SI | AP | C0 | DI | FI |
| --- | --- | --- | --- | --- | --- | --- | --- | --- | --- | --- |
| Age | <40 | 37.88±24.1 | 22.35±26.4 | 31.06±27.37 | 17.42±28.29 | 41.67±28.8 | 29.55±27.10 | 24.24±31.6 | 13.64±23.0 | 53.79  ±37.50 |
|  | 40-49 | 44.64±28.2 | 26.90±31.3 | 42.41±25.14 | 20.47±27.28 | 40.94±30.2 | 43.86±37.35 | 35.09±34.7 | 16.96±28.9 | 60.82  ±36.25 |
|  | 50-59 | 50.06±26.9 | 25.09±32.7 | 43.45±25.57 | 18.73±29.71 | 43.45±30.7 | 43.82±28.69 | 50.19±42.3 | 12.36±27.2 | 68.91  ±32.48 |
|  | 60-69 | 56.36±24.0 | 28.79±28.4 | 54.85±27.34 | 30.30±36.44 | 51.52±30.6 | 50.30±31.99 | 59.39±37.7 | 10.91±24.8 | 73.33±  32.96 |
|  | >70 | 59.06±21.2 | 43.81±34.3 | 61.40±22.94 | 29.82±36.67 | 57.89±29.0 | 61.40±27.80 | 61.40±37.2 | 22.81±31.5 | 71.93±  37.29 |
|  | p-value | 0.002* | 0.126 | 0.000* | 0.128 | 0.102 | 0.002* | 0.000* | 0.434 | 0.037 |
| Marital  status | Single | 28.57±27.4 | 14.29±26.5 | 32.14±31.66 | 14.29±21.54 | 33.33±32.0 | 23.81±33.15 | 16.67±25.3 | 14.29±21.5 | 38.10  ±36.64 |
|  | Married | 48.25±26.1 | 26.57±30.7 | 42.66±25.45 | 21.91±30.92 | 45.45±30.7 | 42.89±31.05 | 39.86±39.4 | 16.78±28.4 | 62.00  ±34.62 |
|  | Divorced | 51.67±31.3 | 33.75±34.0 | 47.08±31.09 | 25.00±35.20 | 45.00±30.7 | 47.50±36.11 | 50.83±41.3 | 9.17±23.85 | 66.67  ±36.20 |
|  | Widowed | 52.57±22.0 | 27.11±29.7 | 51.24±26.16 | 22.39±31.45 | 47.76±29.1 | 48.76±29.14 | 59.70±36.4 | 10.95±25.5 | 79.10  ±30.05 |
|  | p-value | 0.017 | 0.231 | 0.045 | 0.748 | 0.457 | 0.051 | 0.000* | 0.296 | 0.000* |
| Educational status | Illiterate | 51.00±25.6 | 29.91±32.5 | 47.86±28.32 | 24.22±33.51 | 46.72±31.2 | 47.86±30.75 | 57.83±39.2 | 15.38±28.8 | 72.65  ±31.44 |
|  | Can read and write | 46.30±26.3 | 29.86±30.9 | 40.63±23.29 | 18.75±29.09 | 39.58±29.7 | 38.89±31.76 | 43.06±36.3 | 20.83±31.2 | 54.17  ±35.48 |
|  | Informal education | 39.73±20.9 | 19.19±27.0 | 33.33±19.09 | 14.14±27.67 | 47.47±25.0 | 38.38±32.40 | 25.25±32.3 | 13.13±23.4 | 62.63  ±37.96 |
|  | Primary | 46.56±30.9 | 17.46±26.6 | 42.06±26.67 | 19.05±30.86 | 46.03±26.8 | 36.51±33.17 | 23.81±38.2 | 1.59±  7.27 | 50.79  ±38.90 |
|  | Secondary | 48.15±35.6 | 16.67±27.8 | 44.44±34.42 | 16.67±27.88 | 33.33±42.1 | 44.44±40.36 | 33.33±36.5 | 5.56±13.60 | 61.11  ±49.06 |
|  | Collage and above | 54.42±28.7 | 29.06±30.5 | 52.99±30.07 | 29.06±29.79 | 47.86±33.1 | 47.86±33.15 | 41.03±40.0 | 10.26±23.1 | 70.94  ±33.49 |
|  | p-value | 0.220 | 0.270 | 0.030 | 0.354 | 0.651 | 0.340 | 0.000* | 0.096 | 0.010 |
| Occupational status | Government | 53.27±30.3 | 23.53±27.2 | 50.49±30.56 | 26.47±29.33 | 50.98±33.0 | 45.10±31.65 | 38.24±39.4 | 8.82±18.90 | 70.59  ±34.58 |
|  | Private | 34.92±25.1 | 26.98±29.0 | 34.13±30.49 | 22.22±26.52 | 34.92±24.6 | 23.81±23.90 | 22.22±28.5 | 12.70±24.6 | 46.03  ±35.70 |
|  | Merchant | 48.72±27.4 | 17.95±27.0 | 42.31±27.17 | 16.67±28.67 | 44.87±24.8 | 46.15±32.76 | 25.64±34.3 | 8.97±22.22 | 60.26  ±37.73 |
|  | Retired | 53.33±14.4 | 40.00±27.8 | 46.67±34.15 | 40.00±27.88 | 46.67±44.7 | 46.67±44.72 | 53.33±38.0 | 13.33±18.2 | 53.33  ±50.53 |
|  | Farmer | 47.28±21.7 | 32.98±33.0 | 43.26±27.06 | 27.66±32.83 | 40.43±30.2 | 43.26±31.78 | 57.45±37.8 | 21.28±33.6 | 64.54  ±35.71 |
|  | Housewife | 50.90±26.7 | 27.78±32.0 | 46.64±25.37 | 19.64±32.43 | 47.55±31.1 | 47.55±31.67 | 50.90±40.1 | 14.21±27.2 | 69.51  ±33.08 |
|  | Unemployed | 11.11±15.7 | .00 | 25.00±11.78 | .00 | 33.33+.00 | .00 | .00 | .00 | 83.33  ±23.57 |
|  | p-value | 0.062 | 0.331 | 0.343 | 0.384 | 0.441 | 0.028 | 0.000* | 0.3999 | 0.100 |
| Monthly income | <6000ETB | 45.69±25.1 | 26.95±31.4 | 42.68±26.42 | 18.07±29.41 | 45.17±29.0 | 42.37±30.90 | 49.84±39.7 | 15.58±29.4 | 67.91  ±34.54 |
|  | ≥6000ETB | 50.96±27.2 | 27.28±30.6 | 46.50±27.60 | 24.84±32.22 | 45.44±31.3 | 45.22±32.68 | 42.25±39.4 | 12.95±24.9 | 64.33  ±35.42 |
|  | p-value | 0.113 | 0.931 | 0.263 | 0.084 | 0.945 | 0.477 | 0.127 | 0.436 | 0.416 |

FA: fatigue, NV: nausea and vomiting, PA: pain, DY: dyspnea, SI: insomnia, AP: appetite loss, CO: constipation, DI: diarrhea, FI: financial difficulty, ETB: Ethiopian birr

**This is S4 Table: Analysis of the mean differences in EORTC QLQ-C30 symptom scale with clinical characteristics using one way ANOVA, Aug 2021**.

| Variables | Category | FA | NV | PA | DY | SL | AP | CO | DI | FI |
| --- | --- | --- | --- | --- | --- | --- | --- | --- | --- | --- |
| Patient status | New | 45.89±24.8 | 25.64±30.11 | 41.59±25.16 | 19.55±29.1 | 44.23±29.1 | 43.59±30.76 | 43.1±39.12 | 13.62  ±25.8 | 62.34  ±35.09 |
|  | Follow up | 59.72±29.7 | 32.74±33.32 | 57.44±30.63 | 31.55±36.7 | 49.40±34.8 | 45.83±+36.27 | 53.57±+41.03 | 15.48±30.4 | 78.57  ±32.05 |
|  | p-value | 0.000* | 0.127 | 0.000* | 0.010 | 0.259 | 0.642 | 0.080 | 0.647 | 0.002* |
| Time since diagnosis | <1yr | 46.82±25.7 | 24.62±29.61 | 42.68±+26.52 | 20.64±30.2 | 43.51±29.9 | 41.98±31.15 | 44.07±39.85 | 13.25±26.2 | 64.85  ±35.25 |
|  | 1-5yrs | 66.67±26.0 | 50.00±32.96 | 65.28±23.00 | 33.33±35.4 | 61.11±28.9 | 62.50±33.06 | 58.33±37.10 | 22.22±32.1 | 73.61  ±32.57 |
|  | p-value | 0.000* | 0.000* | 0.000* | 0.007 | 0.005* | 0.002* | 0.234 | 0.258 | 0.315 |
| Stage of cancer | Stage I | 30.62±24.4 | 21.14±29.58 | 26.02±19.73 | 14.63±26.9 | 33.33±27.8 | 26.83±30.93 | 26.83±31.81 | 8.94+16.70 | 43.09  ±34.35 |
|  | Stage II | 40.58±22.7 | 18.30±26.38 | 33.88±22.29 | 16.30±27.2 | 37.68±28.4 | 36.96±29.42 | 31.88±36.61 | 12.68±25.6 | 56.88  ±34.42 |
|  | Stage III | 56.63±23.5 | 30.74±+32.23 | 55.02±24.45 | 19.74±29.6 | 51.13±29.0 | 50.16±29.84 | 56.31±39.06 | 16.18±29.6 | 77.99  ±29.72 |
|  | Stage IV | 73.81±22.2 | 51.79±26.96 | 72.02±23.59 | 60.71±28.7 | 66.67±30.0 | 70.24±27.72 | 76.19±31.23 | 17.86±30.7 | 83.33  ±30.76 |
|  | P-value | 0.000* | 0.000* | 0.000* | 0.000* | 0.000* | 0.000* | 0.000* | 0.403 | 0.000* |
| Treatment plan | Surgery | 34.54±22.6 | 21.38±29.37 | 29.17±20.01 | 13.77±24.2 | 34.78±28.3 | 33.70±32.59 | 30.07±34.96 | 9.42±17.34 | 50.00  ±35.46 |
|  | Chemo | 55.97±23.7 | 19.75±+19.63 | 54.32±24.71 | 32.10±31.3 | 46.91±26.5 | 39.51±32.07 | 29.63±37.36 | 18.52±33.7 | 67.90  ±31.32 |
|  | Rad | 72.22±39.2 | 66.67±47.14 | 66.67±47.14 | 66.67±.000 | 50.00±23.5 | 66.67±47.14 | 66.67±47.14 | 83.33±23.5 | 83.33  ±23.57 |
|  | Chemo-rad | 57.11±25.8 | 33.09±32.58 | 54.17±27.04 | 26.23±34.2 | 51.23±31.4 | 52.21±29.73 | 60.29±37.95 | 15.93±29.5 | 76.47  ±31.46 |
|  | Chemo-surgery | 41.27±8.39 | 4.76±12.59 | 30.95±14.99 | .00 | 61.90±12.5 | 33.33±19.24 | 9.52±16.26 | .00 | 52.38  ±37.79 |
|  | p-value | 0.000* | 0.002* | 0.000* | 0.001* | 0.000* | 0.000* | 0.000* | 0.001* | 0.000* |
| Comorbid  conditions | None | 27.04±27 | 25.71±30.90 | 44.02±27.01 | 20.78±30.7 | 43.80±30.6 | 44.10±32.47 | 44.69±40.52 | 12.41±25.5 | 65.62  ±34.57 |
|  | HTN | 21.9±21.98 | 37.30±25.76 | 50.79±29.09 | 31.75±34.1 | 44.44±28.5 | 36.51±27.69 | 46.03±35.70 | 22.22±32.2 | 57.14  ±41.01 |
|  | DM | 16.68±16.68 | 28.79±32.56 | 48.48±22.91 | 27.27±32.7 | 60.61±13.4 | 42.42±26.20 | 39.39±35.95 | 24.24±33.6 | 69.70  ±34.61 |
|  | Cardiac | 15.71±15.71 | 25.00±33.35 | 25.00±11.78 | 16.67±23.57 | 50.00±23.57 | 50.00±23.57 | 66.67±47.14 | 16.67±23.57 | 100.00 |
|  | Renal | - | 100.00 | 100.00 | 66.67 | 66.67 | 100.00 | 100.00 | 100.00 | 100.00 |
|  | Others | 32.71±32.71 | 30.56±34.02 | 50.00±29.81 | 22.22±40.36 | 72.22±38.96 | 61.11±32.77 | 61.11±25.09 | 11.11±17.21 | 77.78  ±34.42 |
|  | p-value | 0.301 | 0.133 | 0.234 | 0.443 | 0.125 | 0.303 | 0.592 | 0.011 | 0.421 |

FA: fatigue, NV: nausea and vomiting, PA: pain, DY: dyspnea, SL: insomnia, AP: appetite loss, CO: constipation, DI: diarrhea, FI: financial difficulty, HTN: hypertension, DM: diabetes.

**This is S5 Table: Analysis the mean differences in** **EORTCQLQ-CX24 functional scale with socio-demographic characteristics using one way ANOVA, Aug 2021**.

| Variables | Category | Sexual/vaginal function | Body Image | Sexual enjoyment | Sexual activity |
| --- | --- | --- | --- | --- | --- |
| Age | <40 | 39.24±19.57 | 40.91±29.32 | 26.39±31.05 | 24.24±26.28 |
|  | 40-49 | 39.17± 22.96 | 42.50± 31.99 | 21.11±28.34 | 26.32±30.69 |
|  | 50-59 | 35.19± 17.51 | 35.83±30.51 | 22.22±22.86 | 10.49± 24.41 |
|  | 60-69 | 56.67± 27.25 | 35.56± 26.91 | 33.33±25.57 | 4.24± 14.42 |
|  | >70 | 33.33± 13.94 | 44.44±30.76 | 33.33± 36.51 | 19.30±33.91 |
|  | p-value | 0.713 | 0.012 | 0.854 | 0.000* |
| Marital status | Single | 31.67±13.69 | 42.06±34.09 | 20.00±29.81 | 11.90±16.58 |
|  | Married | 39.02±22.64 | 41.10±29.84 | 23.28±29.11 | 22.61±30.28 |
|  | Divorced | 38.33±11.18 | 36.11±27.42 | 40.00±14.91 | 5.00±14.22 |
|  | Widowed | 42.50±14.41 | 34.33±30.63 | 26.67±26.29 | 7.46±20.78 |
|  | p-value | 0.828 | 0.420 | 0.616 | 0.000* |
| Education | Illiterate | 36.94±21.52 | 32.76±29.03 | 27.78±23.29 | 14.81±29.51 |
|  | Can read and write | 36.76±21.05 | 28.70±24.78 | 21.57±26.19 | 15.28±23.78 |
|  | Informal education | 41.11±22.59 | 52.86±33.68 | 26.67±40.24 | 21.21±26.11 |
|  | Primary | 38.10±22.49 | 45.50±29.59 | 23.81±37.09 | 17.46±27.12 |
|  | Secondary | 38.89±12.73 | 38.89±34.96 | 11.11±19.25 | 27.78±38.97 |
|  | Collage and above | 45.45±18.77 | 52.99±25.29 | 21.21±22.47 | 10.26±17.36 |
|  | p-value | 0.893 | 0.000* | 0.917 | 0.482 |
| Occupation | Government | 40.38±16.61 | 51.31±27.89 | 23.08±31.58 | 14.81±29.51 |
|  | Private | 37.88±19.85 | 38.62±32.32 | 21.21±22.47 | 15.28±23.78 |
|  | Merchant | 50.93±16.38 | 50.85±26.69 | 22.22±23.57 | 21.21±26.11 |
|  | Retired | 41.67±14.43 | 44.44±17.57 | 44.44±50.92 | 17.46±27.12 |
|  | Farmer | 40.28±31.60 | 30.73±27.34 | 20.37±25.92 | 27.78±38.97 |
|  | Housewife | 33.91±15.09 | 35.06±30.41 | 27.59±29.64 | 10.26±17.36 |
|  | Unemployed | 0 | 72.22±23.57 | 0 | 14.81±29.51 |
|  | P-value | 0.433 | 0.005* | 0.787 | 0.169 |
| Monthly income | <6000ETB | 32.69±21.46 | 30.43±29.01 | 30.77±28.16 | 13.71±28.21 |
|  | ≥6000ETB | 41.81±19.951 | 44.30±29.289 | 21.64±27.811 | 16.77±25.493 |
|  | p-value | 0.063 | 0.000* | 0.171 | 0.359 |

**This is S6 Table: Analysis of the mean differences in EORTC QLQ-CX24 functional scales with clinical characteristics using one way ANOVA, Aug 2021**.

| Variables | Category | Sexual/vaginal function | Body Image | Sexual enjoyment | Sexual activity |
| --- | --- | --- | --- | --- | --- |
| Patient status | New | 38.77±20.61 | 36.49±28.98 | 24.07±27.53 | 16.99±27.22 |
|  | Follow up | 40.15±22.61 | 46.83±32.11 | 27.27±32.72 | 10.12±23.72 |
|  | P-value | 0.839 | 0.021 | 0.727 | 0.087 |
| Time since diagnosis | <1yr | 38.40±20.95 | 36.17±29.45 | 24.47±28.59 | 16.32±26.96 |
|  | 1-5yrs | 50.00±13.61 | 61.11±22.46 | 25.00±16.67 | 8.33±22.52 |
|  | p-value | 0.278 | 0.000* | 0.971 | 0.317 |
| Stage of cervical ca | Stage I | 35.83±14.33 | 30.89±29.66 | 20.00±19.94 | 23.58±29.10 |
|  | Stage II | 38.28±18.31 | 31.28±28.99 | 28.13±32.91 | 15.22±24.42 |
|  | Stage III | 35.14±24.61 | 43.58±29.14 | 24.64±28.81 | 12.30±25.98 |
|  | Stage IV | 60.42±22.16 | 56.35±26.00 | 20.83±24.80 | 16.67±30.77 |
|  | p-value | 0.017 | 0.000* | 0.763 | 0.149 |
| Treatment plan | Surgery | 36.11±17.66 | 30.92±29.18 | 23.93±27.52 | 21.01±29.12 |
|  | Chemo | 52.78±26.35 | 37.86±37.14 | 25.93±36.43 | 13.58±21.20 |
|  | Rad | 33.33 | 61.11±39.28 | 33.33 | 16.67±23.57 |
|  | Chemo-rad | 37.50±21.89 | 43.71±27.79 | 26.04±27.74 | 12.50±25.96 |
|  | Chemo- surgery | 58.33±11.79 | 39.68±31.98 | .00 | 9.52±16.27 |
|  | p-value | 0.150 | 0.023 | 0.789 | 0.186 |
| Comorbid  Conditions | None | 39.34±21.83 | 37.77±28.84 | 22.55±26.67 | 15.25±26.79 |
|  | HTN | 35.42±16.33 | 40.74±35.89 | 30.56±30.01 | 26.98±29.09 |
|  | DM | 33.33 | 45.45±37.00 | 33.33 | 3.03±10.05 |
|  | Cardiac | 00 | 27.78±39.28 | 00 | 33.33±47.14 |
|  | Renal | 50.00 | 33.33 | 00 | 00 |
|  | Others | 50.00 | 57.41±36.12 | 100.00 | 5.56±13.61 |
|  | p-value | 0.913 | 0.624 | 0.057 | 0.134 |

Rad: radiation, Chem: chemotherapy

**This is S7 Table: Analysis of the mean difference in EORTC QLQ-CX24 symptom scales with socio-demographic characteristics using one way ANOVA, Aug 2021**.

| Variables | Category | SE | LY | PN | MS | SXW |
| --- | --- | --- | --- | --- | --- | --- |
| Age | <40 | 24.65±16.25 | 21.21±32.22 | 19.70±30.76 | 29.55±35.37 | 37.12± 38.18 |
|  | 40-49 | 35.03±18.82 | 23.98±34.93 | 31.58±36.41 | 33.33±35.07 | 43.27± 32.71 |
|  | 50-59 | 35.85± 17.22 | 19.10±30.93 | 22.47±29.63 | 38.95±38.34 | 25.09± 35.63 |
|  | 60-69 | 36.80± 14.85 | 27.88±33.10 | 26.67±31.69 | 46.06± 41.81 | 17.58± 32.61 |
|  | >70 | 48.48± 17.69 | 49.12±44.95 | 47.37±40.54 | 35.09± 39.24 | 29.82±36.67 |
|  | p-value | 0.000* | 0.000* | 0.078 | 0.215 | 0.000* |
| Marital status | Single | 18.18±12.47 | 14.29±31.25 | 16.67±31.35 | 14.29±21.54 | 35.71±35.72 |
|  | Married | 34.44±18.39 | 21.91±32.16 | 27.27±33.25 | 37.30±37.21 | 35.20±37.89 |
|  | Divorced | 33.94±15.13 | 30.00±37.59 | 23.33±31.31 | 35.83±38.78 | 27.50±32.81 |
|  | Widowed | 40.03±16.76 | 28.86±36.65 | 29.35±34.58 | 43.28±41.04 | 18.41±30.86 |
|  | p-value | 0.000* | 0.243 | 0.541 | 0.077 | 0.014 |
| Education  status | Illiterate | 38.49±17.50 | 21.65±33.42 | 29.91±35.66 | 36.75±35.12 | 20.23±30.94 |
|  | Can read and write | 34.22±18.14 | 26.39±32.95 | 29.86±32.43 | 33.33±38.89 | 23.61±32.95 |
|  | Informal education | 27.55±16.59 | 19.19±28.90 | 19.19±28.90 | 28.28±36.44 | 53.54±38.13 |
|  | Primary | 25.54±17.21 | 22.22±38.49 | 25.40±36.37 | 22.22±33.88 | 52.38±40.24 |
|  | Secondary | 36.36±16.82 | 27.78±44.31 | 22.22±40.37 | 50.00±45.95 | 38.89±38.97 |
|  | Collage and above | 36.13±16.95 | 35.90±37.76 | 20.51±26.06 | 58.12±41.69 | 32.48±35.45 |
|  | p-value | 0.005* | 0.286 | 0.453 | 0.003* | 0.000* |
| Occupational status | Government | 33.24±17.87 | 31.37±36.65 | 18.63±27.45 | 49.02±40.39 | 39.22±38.02 |
|  | Private | 25.97±17.60 | 20.63±35.71 | 23.81±31.87 | 34.9±38.69 | 41.27±34.81 |
|  | Merchant | 29.49±17.47 | 15.38±28.65 | 14.10±26.95 | 34.62±44.70 | 39.74±37.74 |
|  | Retired | 49.09±19.69 | 86.67±29.81 | 53.33±38.01 | 40.00±54.77 | 20.00±18.26 |
|  | Farmer | 41.01±21.79 | 21.28±32.17 | 34.75±39.29 | 38.30±33.32 | 17.73±27.67 |
|  | Housewife | 35.54±15.00 | 24.29±33.27 | 28.17±32.39 | 35.40±37.21 | 27.39±36.67 |
|  | Unemployed | 9.09±4.29 | .00 | .00 | .00 | 83.33±23.57 |
|  | P-value | 0.001* | 0.001* | 0.034 | 0.459 | 0.008 |
| Monthly income | <6000ETB | 34.64±17.25 | 17.13±29.80 | 22.74±31.26 | 32.09±36.61 | 23.05±33.46 |
|  | ≥6000ETB | 35.11±18.21 | 29.51±36.19 | 29.30±34.25 | 40.98±38.84 | 34.39±36.87 |
|  | p-value | 0.833 | 0.004* | 0.115 | 0.003* | 0.011 |

SE: symptom experience, LY: Lymph edema, PN: Peripheral neuropathy, MS: Menopausal symptoms, SXW: Sexual worry

**This is S8 Table: Analysis of the mean differences in EORTC QLQ-CX24 Symptom scales with clinical characteristics using one way ANOVA, Aug 2021**.

| Variables | Category | SE | LY | PN | MS | SXW |
| --- | --- | --- | --- | --- | --- | --- |
| Patient status | New | 33.28±18.12 | 20.99±32.31 | 26.28±34.11 | 30.29±35.23 | 31.09±35.82 |
|  | Follow up | 41.02±15.16 | 37.50±38.17 | 27.98±29.66 | 63.69±37.21 | 25.00±36.09 |
|  | P-value | 0.004* | 0.001* | 0.735 | 0.000* | 0.261 |
| Time since diagnosis | <1yr | 33.68±17.42 | 21.90±32.62 | 25.10±32.37 | 34.31±36.97 | 29.43±35.68 |
|  | 1-5yrs | 45.71±16.69 | 47.22±39.22 | 40.28±38.04 | 66.67±38.07 | 30.56±36.67 |
|  | p-value | 0.001* | 0.000* | 0.049 | 0.000* | 0.145 |
| Stage of cervical ca | Stage I | 18.70±10.20 | 13.01±26.75 | 18.70±26.93 | 16.26±28.98 | 30.89±36.05 |
|  | Stage II | 29.31±15.07 | 15.94±29.43 | 20.29±29.63 | 29.71±33.31 | 30.80±36.06 |
|  | Stage III | 40.54±14.29 | 26.54±32.46 | 27.51±33.79 | 43.37±38.72 | 26.54±34.73 |
|  | Stage IV | 56.39±16.95 | 61.90±39.25 | 55.95±35.19 | 71.43±35.96 | 36.90±39.89 |
|  | p-value | 0.000* | 0.000* | 0.000* | 0.000* | 0.563 |
| Treatment plan | Surgery | 22.99±12.71 | 11.96±27.33 | 15.94±26.83 | 21.01±31.14 | 32.25±36.13 |
|  | Chemo | 38.27±13.29 | 16.05±26.75 | 18.52±26.69 | 28.40+34.22 | 32.10±36.38 |
|  | Rad | 48.48±4.29 | 83.33±23.57 | 83.33±23.57 | 83.33±23.57 | 16.67±23.57 |
|  | Chemo-Rad | 42.71±17.22 | 34.80±36.49 | 35.78±35.52 | 50.49±38.27 | 26.47±34.93 |
|  | Chemo -Surgery | 23.38±12.58 | 4.76±12.59 | 4.76±12.59 | 19.05±37.79 | 57.14±46.00 |
|  | p-value | 0.000* | 0.000* | 0.000* | 0.000* | 0.196 |
| Comorbid conditions | None | 34.05+17.84 | 22.42+32.96 | 23.32+30.58 | 36.32+37.44 | 28.85+35.09 |
|  | HTN | 40.55±19.43 | 38.10±38.42 | 50.79±40.30 | 47.62±38.83 | 31.75±35.71 |
|  | DM | 40.50±15.82 | 21.21±34.23 | 27.27±35.96 | 18.18±31.14 | 39.3±46.71 |
|  | Cardiac | 45.45±12.86 | 66.67±47.14 | 83.33±23.57 | 50.00±70.71 | 33.33±47.14 |
|  | Renal | 45.45 | 100.00 | 66.67 | 100.00 | .00 |
|  | others | 31.82±2.38 | 33.33±42.16 | 38.89±49.07 | 61.11±49.06 | 44.44±50.19 |
|  | p-value | 0.425 | 0.027 | 0.000* | 0.082 | 0.748 |

SE: symptom experience, LY: Lymph edema, PN: Peripheral neuropathy, MS: Menopausal symptoms, SXW: Sexual worry
